# Technical note: optimizing and validating an RP-HPLC method to determine lactoferrin in porcine colostrum and milk

**DOI:** 10.1093/jas/skaf068

**Published:** 2025-03-07

**Authors:** Katharina Metzger, Ulrike Gimsa, Winfried Otten

**Affiliations:** Research Institute for Farm Animal Biology (FBN), 18196 Dummerstorf, Germany; Research Institute for Farm Animal Biology (FBN), 18196 Dummerstorf, Germany; Research Institute for Farm Animal Biology (FBN), 18196 Dummerstorf, Germany

**Keywords:** animal health, antimicrobials, colostrum, milk, pig

## Abstract

This Technical Note provides a detailed description of a sample preparation procedure, along with the validation of a reversed-phase high-performance liquid chromatography (RP-HPLC) method for quantitatively determining lactoferrin (LF) in porcine colostrum and milk. The analysis of native milk samples is a challenging process due to the complex composition of the sample. Raw milk is an emulsion and colloid of fat globules in a water-based liquid containing dissolved carbohydrates and protein aggregates with minerals. This paper aims to optimize a method for preparing porcine colostrum and milk samples, which involves a new combination of homogenization, centrifugation, dilution, and filtration techniques for the subsequent analysis of LF via RP-HPLC. A validation process was implemented to ensure the reliability and repeatability of this analytical approach. The results showed that the quantification of LF content in porcine colostrum (1,684.58 ± 466.68 µg/mL; *n* = 10) and milk (849.75 ± 85.82 µg/mL; *n* = 10) is feasible with successful validation. In addition, the improved sensitivity due to the novel combination of these preparation steps is shown by low limits of detection and quantification of 0.21 µg/mL each. The advantage of this optimized methodology is that the sample preparation can be carried out relatively simply and using standard laboratory equipment, thus enabling the accurate determination of LF in porcine colostrum and milk samples.

## Teaser

This optimized methodology for porcine colostrum and milk samples is relatively simple to implement and enables the accurate determination of lactoferrin via reversed-phase high-performance liquid chromatography.

## Introduction

Lactoferrin (LF) is an 80-kDa iron-binding glycoprotein, initially identified as a protein present in the whey of bovine milk. It was subsequently purified as a major iron-binding glycoprotein in human and bovine milk (Sorensen and Sorensen, 1939; Groves, 1960; Johanson, 1960). The amino acid sequences of LF from humans, cows, and pigs are 70% identical (Lydon et al., 1992). Furthermore, LF is found in secretions such as milk, tears, saliva, gastric juice, and semen (González-Chávez et al*.,* 2009; Rosa et al., 2017). However, the highest LF concentrations are found in colostrum followed by milk where LF is part of the whey proteins. There are large species-specific differences. The highest LF content is found in human milk (1.5 to 2.0 g/L), whereas lower contents are found in equine (0.2 to 2.0 g/L), bovine (0.1 to 1.5 g/L), donkey (0.08 to 0.25 g/L), buffalo (0.03 to 0.23 g/L), camel (0.02 to 0.22 g/L), and goat (0.02 to 0.2 g/L) milk (reviewed in Hong et al., 2024). LF is an important host defense molecule with protective effects against bacteria, fungi, viruses, parasites, and has antitumor activity, as well as other activities related to inflammatory responses and immune function (Wakabayashi et al., 2000; van der Strate et al., 2001; González-Chávez et al., 2009). Consequently, at the onset of the COVID-19 pandemic, LF was identified as a potential agent for the prevention or adjunctive treatment of the disease’s symptoms (Einerhand et al., 2022).

From an immunological perspective, rapid colostrum intake is of the utmost importance for newborns (Heuß et al., 2019). Especially in piglets, colostrum serves as the primary source of passive immunity, as the placenta prevents the transfer of maternal antibodies and leukocytes to the fetus during gestation (Pettigrew, 1981; Rooke and Bland, 2002). It contains many growth factors, immunoglobulins, and bioactive components such as LF (Theil et al., 2014). The amount of LF in colostrum is higher than in milk and other body fluids (Elliot et al., 1984; González-Chávez et al., 2009). The necessity for a reliable method of determining the concentration of LF in sow colostrum and milk has arisen from the increasing commercial interest in its therapeutic value and in the rearing of vital and healthy piglets. In pig rearing, maintaining a high survival rate in newborn piglets remains a major challenge (Indyk and Filonzi, 2005; Heuß et al., 2019).

There are a variety of methods for the determination of milk proteins. These include electrophoresis techniques, enzyme-linked immunosorbent assays (ELISA), and high-performance liquid chromatography (HPLC). The determination by HPLC is suitable for separating milk proteins, and a high resolution and accuracy are ensured by additional reversed-phase (RP) methodology (Bonfatti et al., 2008; Masci et al., 2022). HPLC methods for determining LF in milk have already been developed for a variety of farm animals, including cattle (Pochet et al., 2018), goats (Drackova et al., 2009), sheep (Tsakali et al., 2019), and pigs (Jahan et al., 2020). The instrument sensitivity of the HPLC methods was determined by applying the commonly used 3×signal/noise ratio (3 S/N) for the limit of detection (LOD) and 10×signal/noise ratio (10 S/N) for the limit of quantification (LOQ). The LODs at 3 S/N show great variation such as 2 µg/mL (Pochet et al., 2018), 4.5 µg/mL (Drackova et al., 2009), 35.45 µg/mL Tsakali et al. (2014)—this method was used for the samples determined in Tsakali et al. (2019)—, and 1 µg/mL (Jahan et al., 2020). Whereas, the LODs at 10 S/N were 7 µg/mL (Pochet et al., 2018), 15 µg/mL (Drackova et al., 2009), 50.86 µg/mL (Tsakali et al., 2014)—this method was used for the samples determined in Tsakali et al, (2019)—, and 5 µg/mL (Jahan et al., 2020). The work of Jahan et al. (2020) describes the analysis of LF from porcine colostrum and milk using RP-HPLC. However, we had difficulties in successfully reproducing this method. This might be due to the complex composition of such emulsions, which requires appropriate preparation, including homogenizing the samples and centrifuging the sample several times at the appropriate sedimentation rate, followed by dilution and filtration. Minimizing the viscosity of the sample and the fat content is crucial, as these are well-established methods for preparing dairy products (Strum et al., 2018; Tsakali et al., 2019; Mangione et al., 2022; von Oesen et al., 2023). These studies also showed that accurate analysis is impossible without any appropriate sample preparation. Consequently, the study aims to establish and validate an optimized analytical methodology, including appropriate sample preparation by a novel combination of preparation steps and with standard lab equipment, to analyze LF in porcine colostrum and milk by RP-HPLC.

## Material and Methods

### Sample collection

This study did not require a review and approval process in accordance with ethical standards, as the samples were obtained in the context of the routine health monitoring of breeding stock at the Research Institute for Farm Animal Biology’s (FBN) experimental pig facility.

Porcine colostrum and milk samples of approximately 2 to 5 mL were collected in sterile tubes by manual milking during milk letdown at two time points of lactation. The samples were taken from a middle teat and from the right or left side, depending on availability. Samples were collected after milk letdown in a 10- to 20-s time window initiated by the piglets’ suckling stimulus. Colostrum samples were collected during parturition (*n* = 10), and milk samples on the seventh day of lactation (*n* = 10). All the samples were collected during a consistent period, between 07:00 and 10:00, and then stored at −20 °C until analysis.

### Sample preparation

After thawing the colostrum and milk samples, homogenization was performed using an Ultra-Turrax 25 homogenizer (IKA-Werke, Staufen, Germany) twice for 30 s each at 8,000 rpm. Subsequently, samples were centrifuged at 4,000 × g for 15 min at 4 °C, after which the supernatants were placed in precooled tubes. All subsequent work was conducted on ice, with the required plastic equipment and fluids being precooled in advance. In the next step, the casein in the sample was precipitated by adjusting the pH to 4.6 with 1 mol/L HCl (Carl Roth, Karlsruhe, Germany). Subsequently, the samples were centrifuged at 4,000 × g for 15 min at 4 °C. The supernatants were transferred to a tube, and diluted with ultrapure water. Colostrum samples were diluted 1:5, while milk samples were diluted 1:2.5 and centrifuged at 4,000 × g for 15 min at 4 °C to remove any solids. Supernatants were filtered through 15-mm and 0.45-µm cellulose acetate membrane syringe filters (Rotilabo Mini-Tip syringe filter, Carl Roth). The samples prepared were stored at −20 °C until analyzed by RP-HPLC.

### LF determination by RP-HPLC

The LF in the supernatant of porcine colostrum or milk whey samples was quantitatively analyzed using a modification of the method described by Osel et al. (2021). For this application, an LC-20 system (Shimadzu, Kyōto, Japan) was equipped with a diode array UV detector (SPD-M20A, Shimadzu) and a column oven (CTO-10AS_VP_, Shimadzu) with a C18 column (ProntoSIL 300-5-C4 EC, 250 × 4.0 mm, Bischoff, Leonberg, Germany). The mobile phase consisted of A: 0.1% trifluoroacetic acid (RotiPuran LC-MS grade, Carl Roth) in 95% ultrapure water and 5% acetonitrile (vol/vol; Sigma-Aldrich, Taufkirchen, Germany) and B: 0.1% trifluoroacetic acid (RotiPuran LC-MS grade, Carl Roth) in 5% ultrapure water and 95% acetonitrile (vol/vol; Sigma-Aldrich) at a flow rate of 1 mL/min and an oven temperature of 35 °C. The following gradient program was used: 0.00 to 16.00 min 25% to 44% B, 16.01 to 20.00 min 44% to 95% B, and 20.01 to 45.00 min 95% to 25% B. The injection volume was 40 µL, and the autosampler had a constant temperature of 4 °C. Absorbance was measured at a wavelength of 280 nm. Data acquisition was performed by using the LabSolution Software (Shimadzu). The colostrum and milk LF concentration was quantified by comparison with a five-point standard curve using commercial bovine LF standard (Cerilliant, purity 98.12 wt%, Sigma-Aldrich). All samples were analyzed in triplicate on 3 different days. The final concentration of LF was expressed in µg/mL colostrum or milk.

### Statistical analysis

The linear regression analysis and calculation of the percentage coefficient of variation (CV) for the method validation were performed using MS Excel 2016 (Microsoft, Redmond, WA). The LF concentrations in porcine colostrum and milk were presented as mean ± standard deviation.

## Results and Discussion

**Figure 1.**
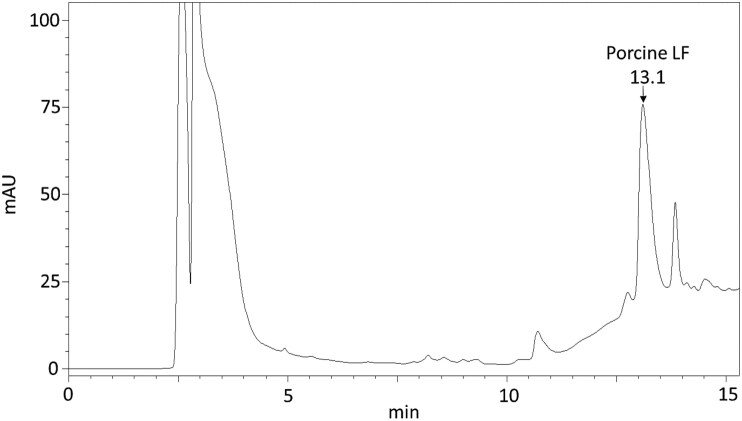
Representative chromatogram of porcine colostrum at 280 nm.

In order to identify the optimal detection wavelength for LF, elution was monitored in the range of 190 to 400 nm conducted on a colostrum sample, a milk sample, and a standard (Figure S1). Detection of LF was achieved at 203 and 277 nm. At 280 nm, the detection of LF had better baseline resolution and less interference to other peaks in the chromatogram, which is consistent with the findings of Osel et al. (2021). The identification of LF in porcine colostrum and milk samples was verified by comparing the UV spectra of the respective peaks with the LF standard. The retention time of the LF standard was 13.2 min, and the peak exhibited a characteristic shape comparable to that observed by Osel et al. (2021) and Tsakali et al. (2014). Samples were subjected to identical measurement conditions, resulting in LF retention times in colostrum (**Figure 1**) and milk between 13.1 and 13.2 min (Figure S2). The chromatogram of porcine colostrum displayed a resolved peak that did not interfere with other peaks, and similar chromatograms were found for porcine milk. In addition, peak symmetry was verified by determining the tailing factor (TF). Peak tailing in biological compounds partially results from the interaction between metal ions (for example, Fe^2+/3+^) on the surface on the LC systems, hindering the separation of analytes (Abrahamson et al., 1994). Thus, in case of LF, which is an iron-containing protein, the determination of the TF is of great relevance. According to Wang et al. (2025), a reliable TF is <2 for an analyte peak. In our study, the TF for the colostrum and milk samples were 1.895 and 1.998, respectively, fulfilling this criterion. LF peaks in the milk of other species often show interference with anionic and cellular components due to the high isoelectric point (pI >8; Pochet et al., 2018; Tsakali et al., 2019).

To assess the efficacy of this methodology, its specificity, linearity, sensitivity, repeatability, recovery rate, and intra- and interday variation were evaluated. The specificity of the method was checked by comparing the retention time of the bovine milk LF standard, milk, and water (sample dilution). Peak purity was assessed based on the degree of similarity of the UV spectra across the peak in the range of 200 to 800 nm and expressed as peak purity index. The peak purity index was 0.999 for all samples, which was considered acceptable to demonstrate the spectral homogeneity of the peak (Karthikeyan et al., 2009). A standard curve was constructed using five different LF concentrations in the range of 6.25 to 100 µg, and measured four times to validate the linearity of the method. The standard curve’s slope, intercept, and linearity were all evaluated by linear regression analysis, with the resulting *R*² value being higher than 0.999. The instrument sensitivity was characterized by the LOD at 3 S/N being 0.21 µg/mL and the LOQ at 10 S/N also being 0.21 µg/mL. Both values were lower than those of Jahan et al. (2020) and Drackova et al. (2009), indicating an improved sensitivity of our method. The repeatability of the method was evaluated by calculating the percentage CV of samples with low (milk) and high (colostrum) LF concentrations. To this end, each sample was measured in triplicate for 3 d. The CV ranged from 3.1% (milk) to 4.9% (colostrum), demonstrating that the method was reproducible. Furthermore, for recovery studies, five milk samples were spiked with three different LF concentrations (2.5, 5, and 7.5µg) and measured as duplicates. The mean recovery rate for the spiked amount of 2.5 µg was 92.72% ± 1.98%, for the spiked amount of 5.0 µg, 86.85% ± 2.92%, and for the spiked amount of 7.5 µg, 82.42% ± 3.54% (for detailed information, see Table S1). The results of the recovery studies indicate that the presence of another peak for porcine LF is unlikely to have interfered with the results. For the intraday and interday assays, a single pooled sample was established from the colostrum of five sows. Twelve measurements of the pooled sample were conducted on a single day to determine intraday variation. In contrast, the pooled sample was measured in triplicate over a period of 15 d to reveal the interday variation. The precision of the assays was evaluated by calculating the percentage CV. The CV was 3.5% for the intraday assay (Table S2) and 3.2% for the interday assay (Table S3).

**Figure 2.**
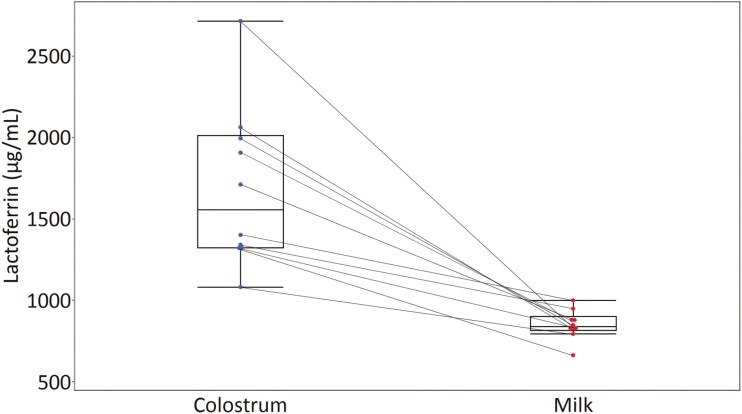
The boxplots illustrate the distribution of lactoferrin content (µg/mL) in 10 colostrum samples collected during parturition and in 10 milk samples from the seventh day of lactation. The values were generated from samples measured in triplicate on 3 different days. The dots in the box plots indicate the mean values for each colostrum and milk sample. The lines connect colostrum and milk samples from the same sow. Boxplots were generated with JMP 15.0.0.

The concentrations of LF (**Figure 2**) observed in porcine colostrum (1,684.58 ± 466.68 µg/mL) and milk on lactation day 7 (849.75 ± 85.82 µg/mL) are comparable to previously reported values. A reduction in LF content is also observed as lactation progresses. This resulted in a natural transition from the initial colostrum to the subsequent milk, accompanied by a shift in composition (Elliot et al., 1984; Yang et al., 2000). However, Jahan et al. (2020) found higher LF levels in porcine colostrum and milk, which may be attributed to genetic differences or differences based on geographical sample origin (Rai et al., 2014). An alternative explanation for this observation could be the suboptimal sample preparation. Samples were centrifuged at 3,000 × g for 15 min at 4 °C before and after whey protein precipitation and then stored at −20 °C until analysis. Achieving phase separation through centrifugation proved challenging when implementing this protocol. Furthermore, the extracts obtained were very turbid, which led to a considerable increase in pressure when loading the HPLC system, ultimately resulting in clogging, which obstructed measurements. Therefore, processing samples from milk and dairy products had to be revised. It was necessary to perform a sequence of preparation steps: (1) homogenization with Ultra-Turrax (Strum et al., 2018; Mangione et al., 2022), (2) centrifugation at higher sedimentation speed (von Oesen et al., 2023), (3) sample dilution (von Oesen et al., 2023), and (4) filtration prior to HPLC analysis (Tsakali et al., 2019). Our approach of sample preparation by combining these four preparation steps is a novelty and showed its advantages in an improved sensitivity of the method with lower LOD and LOQ values compared to published methods of LF analysis by HPLC. Moreover, this protocol could be easily implemented using standard laboratory equipment. The main objectives when rearing pigs are an increased survival rate after birth and healthy piglets before and after weaning. For that reason, the focus of research is increasingly on the protective effects of LF in improving immune functions in young animals. Analytical methods are thus needed to detect LF in sow milk and colostrum and in the development of new functional feed (for example, milk replacers).

In conclusion, the results of our study show that the present novel protocol for sample preparation and quantification of porcine LF by RP-HPLC appears to be a suitable method for determining LF in porcine milk and other samples and can, therefore, be an important component in the development of practices for improving animal health and welfare.

## Supplementary Material

skaf068_suppl_Supplementary_Materials

## Abbreviations

HCl: hydrochloric acid
LC-MS: liquid chromatography–mass spectrometry
LF: lactoferrin
RP-HPLC: reversed-phase high-performance liquid chromatography
