# Supplementary material for: Technical note: optimizing and validating an RP-HPLC method to determine lactoferrin in porcine colostrum and milk
Source: J Anim Sci. 2025 Mar 7;103:skaf068. doi: 10.1093/jas/skaf068 (PMC12010696; doi:10.1093/jas/skaf068)
Supplement: skaf068_suppl_Supplementary_Materials [file skaf068_suppl_supplementary_materials.pdf]

Supplemental Material

Technical Note: Optimizing and Validating an RP-HPLC Method to Determine Lactoferrin in Porcine Colostrum and Milk

Katharina Metzger*, Ulrike Gimsa, Winfried Otten

Research Institute for Farm Animal Biology (FBN), 18196 Dummerstorf, Germany

* Corresponding author: metzger@fbn-dummerstorf.de (K. Metzger)

Research Institute for Farm Animal Biology (FBN), 18196 Dummerstorf, Germany, tel.: +49 38208 68810, fax: +49 38208 68802

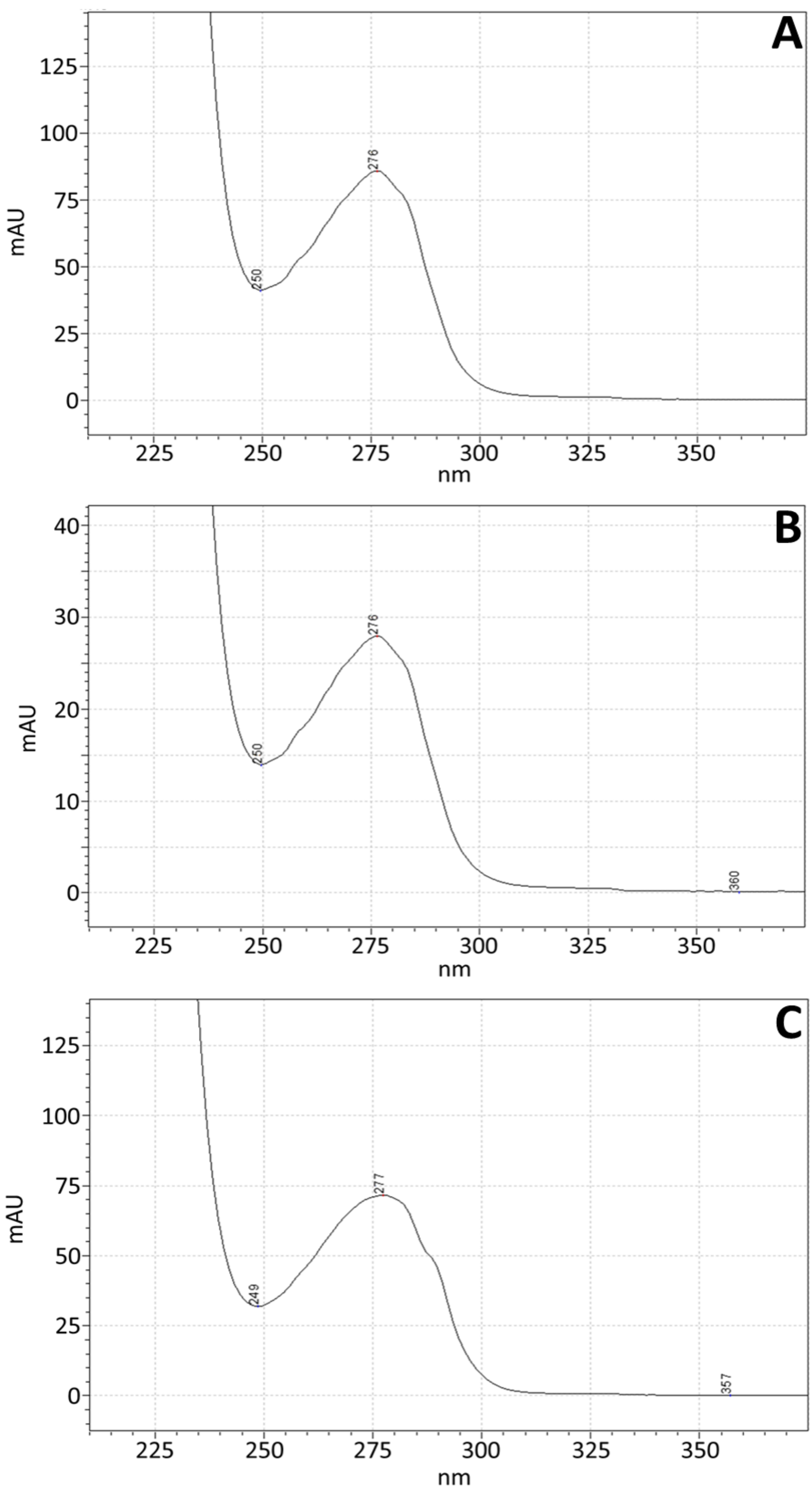

Figure S1. UV spectra of (A) a porcine colostrum, (B) porcine milk from the seventh day of lactation and (C) a bovine lactoferrin standard (25 µg/mL).

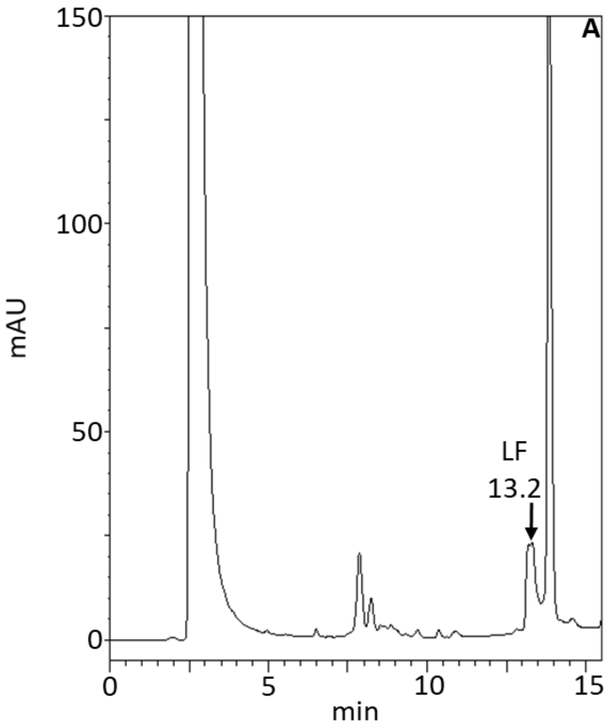

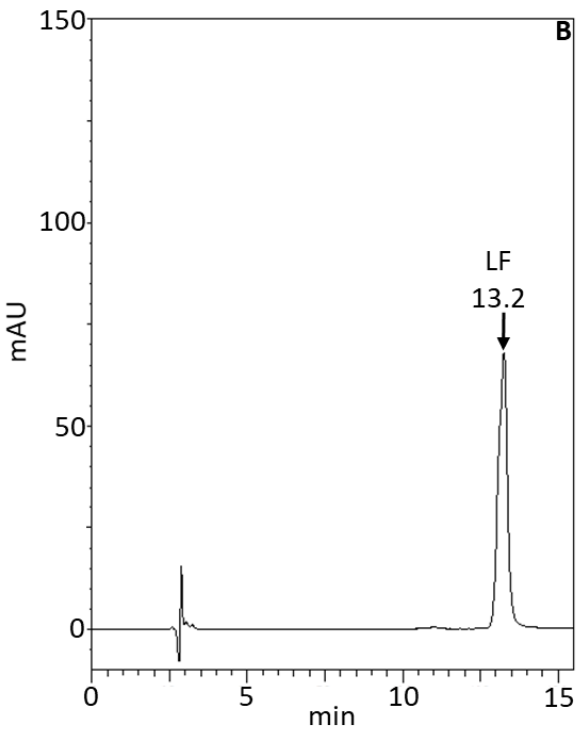

Figure S2. Representative chromatograms of (A) a porcine milk sample from the seventh day of lactation and (B) a bovine lactoferrin standard (25 µg/mL) at 280 nm.

Table S1 – Overview of Recovery studies

**Spiked Sample**	**Recovery rate (%)**		
	**Spike 2.5 µg**	**Spike 5.0 µg**	**Spike 7.5 µg**
**1**	93.00 ± 0.26	88.84 ± 0.91	84.79 ± 2.50
**2**	96.44 ± 0.65	91.08 ± 1.06	88.11 ± 0.34
**3**	91.58 ± 2.29	86.34 ± 2.09	80.39 ± 0.45
**4**	90.83 ± 0.04	85.40 ± 0.26	80.49 ± 0.35
**5**	91.73 ± 0.50	82.56 ± 0.26	78.31 ± 1.35
**Mean ± SD**	**92.72 ± 1.98**	**86.85 ± 2.92**	**82.42 ± 3.54**
**CV**	**2.1**	**3.4**	**4.3**

SD – standard deviation, CV – percentage coefficient of variation

Table S2 – All measured values of the intra-day assay

Number of measurement	LF concentration (µg/mL)
**1**	1383.70
**2**	1338.55
**3**	1338.91
**4**	1327.58
**5**	1297.36
**6**	1288.95
**7**	1266.95
**8**	1268.49
**9**	1256.01
**10**	1282.10
**11**	1260.22
**12**	1224.22
**Mean**	**1294.42**
**SD**	**44.91**
**CV**	**3.5**

LF – lactoferrin, SD – standard deviation, CV – percentage coefficient of variation

Table S3 – All values of the inter-day assay

Measuring day	LF concentration (µg/mL, Mean ± SD)
**1**	1329.20 ± 3.30
**2**	1306.17 ± 26.93
**3**	1298.96 ± 14.42
**4**	1393.27 ± 39.41
**5**	1308.99 ± 29.21
**6**	1245.16 ± 52.42
**7**	1316.22 ± 70.78
**8**	1321.08 ± 13.64
**9**	1314.02 ± 39.20
**10**	1307.72 ± 5.80
**11**	1331.98 ± 32.81
**12**	1388.23 ± 17.72
**13**	1355.22 ± 20.26
**14**	1355.73 ± 11.53
**15**	1408.42 ± 19.44
**Mean ± SD**	**1332.02 ± 42.24**
**CV**	**3.2**

LF – lactoferrin, SD – standard deviation, CV – percentage coefficient of variation
